# Impact of the line of treatment on progression-free survival in patients treated with T-DM1 for metastatic breast cancer

**DOI:** 10.1186/s12885-021-08950-x

**Published:** 2021-11-11

**Authors:** A. Migeotte, V. Dufour, A. van Maanen, M. Berliere, J. L. Canon, D. Taylor, F. P. Duhoux

**Affiliations:** 1 Université catholique de Louvain, Brussels, Belgium; 2 Department of Medical Oncology, King Albert II Cancer Institute, Cliniques universitaires Saint-Luc, Brussels, Belgium; 3 Statistical support unit, King Albert II Cancer Institute, Cliniques universitaires Saint-Luc, Brussels, Belgium; 4 Department of Gynecology, King Albert II Cancer Institute, Cliniques universitaires Saint-Luc, Brussels, Belgium; 5 Institut de Recherche Expérimentale et Clinique (Pôle GYNE), Université catholique de Louvain, Brussels, Belgium; 6 Department of Oncology and Hematology, Grand Hôpital de Charleroi, Charleroi, Belgium; 7 Department of Medical Oncology, CHU UCL Namur, site Sainte-Elisabeth, Namur, Belgium; 8 Institut de Recherche Expérimentale et Clinique (Pôle MIRO), Université catholique de Louvain, Brussels, Belgium

**Keywords:** Metastatic breast cancer, Line of treatment, T-DM1, Progression-free survival

## Abstract

**Background:**

Trastuzumab emtansine (T-DM1) is indicated as second-line treatment for human epidermal growth factor receptor 2 (HER2)-positive metastatic or unresectable locally advanced breast cancer, after progression on trastuzumab and a taxane-based chemotherapy.

We wished to determine if the line of treatment in which T-DM1 is administered has an impact on progression-free survival (PFS) and in particular, if prior treatment with capecitabine/lapatinib or pertuzumab modifies PFS of further treatment with T-DM1.

**Patients and methods:**

We performed a multicenter retrospective study in 3 Belgian institutions. We evaluated PFS with T-DM1 in patients treated for HER2 positive metastatic or locally advanced unresectable breast cancer between January 1, 2009 and December 31, 2016.

**Results:**

We included 51 patients. The median PFS was 9.01 months. The line of treatment in which T-DM1 (1st line, 2nd line, 3rd line or 4+ lines) was administered had no influence on PFS (hazard ratio 0.979, CI95: 0.835–1.143).

There was no significant difference in PFS whether or not patients had received prior treatment with capecitabine/lapatinib (9.17 vs 5.56 months, *p*-value 0.875).

But, patients who received pertuzumab before T-DM1 tended to exhibit a shorter PFS (3.55 months for T-DM1 after pertuzumab vs 9.50 months for T-DM1 without pretreatment with pertuzumab), even if this difference was not statistically significant (*p*-value 0.144).

**Conclusion:**

Unlike with conventional chemotherapy, the line of treatment in which T-DM1 is administered does not influence PFS in our cohort of patients with advanced HER2-positive breast cancer.

## Background

Breast cancer is a common disease affecting about one in eight women, with an increasing incidence [1]. During the last decades, the treatment of this disease, which in the beginning was very mutilating, has been perfected and diversified, both at the surgical and at the medical level. Different classes of treatment have been developed: chemotherapy, endocrine therapy and more recently targeted therapies blocking specific receptors.

HER2 receptors are overexpressed in approximately 20% of all breast cancers, and this overexpression is associated with poorer overall survival (OS) [2]. The addition of trastuzumab to first-line therapy showed an OS increase in metastatic disease [3].

In a second step, a new type of targeted therapy has been introduced; trastuzumab emtansine (T-DM1) is a humanized monoclonal antibody targeting human epidermal growth factor receptor 2 (HER2) receptors, conjugated to a cytotoxic component that acts as a microtubule inhibitor [4].

T-DM1 was studied in monotherapy as second-line after the association of trastuzumab with a taxane in HER2-positive locally advanced or metastatic breast cancer and was approved by the United States Food and Drug Administration (FDA) in 2013 [5].

The regulatory approval of this compound was based on the results of the Emilia study, a phase III randomized controlled trial which assessed the PFS and the OS of 991 patients with locally unresectable or metastatic HER2-positive breast cancer treated with T-DM1 vs capecitabine-lapatinib. This trial showed a better PFS (median PFS 9.6 months vs 6.4 months, *p* < 0.001) and a better OS (median OS 29.9 months vs 25.9 months, HR 0.75 (CI95: 0.64–0.88)) with T-DM1 compared to capecitabine-lapatinib, despite 27% of crossover [2, 3].

In HER2-negative metastatic breast cancer, PFS declines with each line of therapy [6], while patients with HER2-positive disease receive more lines of chemotherapy and the longest duration for every line [7]. In our study, we planned to assess whether the line of therapy in which T-DM1 is administered has an impact on PFS.

As capecitabine and lapatinib represented the comparator arm of the Emilia study, none of the patients included in this study had been pretreated with these compounds. The TH3RESA study, another phase III randomized trial, assessed PFS and OS with T-DM1 in comparison with treatment of physician’s choice in 602 advanced HER2-positive breast cancers previously treated with at least trastuzumab, lapatinib and a taxane. This trial showed a superiority of T-DM1, even in third line and beyond (median PFS 6.2 months vs 3.3 months for physician’s choice; median OS 22.7 months vs 15.8 months) [8, 9].

An additional aim of our study was to further analyze the efficacy of T-DM1 after administration of capecitabine and lapatinib in a real world setting.

During the development of T-DM1, the addition of pertuzumab to trastuzumab and taxanes was still under investigation in the first-line treatment of HER2-positive metastatic breast cancer. It ultimately proved its superiority to docetaxel-trastuzumab in PFS and OS (CLEOPATRA study) [5] and has now become standard of care. As a result, in real life, we no longer see the population enrolled in the EMILIA trial [5].

We therefore also wanted to ascertain the efficacy of T-DM1 after the association of taxane-trastuzumab-pertuzumab in daily-life clinical practice, in which most patients are treated in first line with this combination.

## Methods

### Study design and population

This is an observational retrospective study in which we included all patients (male or female) treated at one of the 3 participating institutions who received at least one dose of T-DM1 for the treatment of HER2-positive metastatic or locally advanced inoperable breast cancer, whatever the line of treatment in which T-DM1 was administered. Those patients had been diagnosed with advanced disease between January 1, 2009 and December 31, 2016. They were treated in 3 Belgian institutions: Cliniques universitaires Saint-Luc (CUSL) in Brussels, CHU UCL Namur (Sainte-Elisabeth) (CMSE) in Namur, and Grand Hôpital de Charleroi (GHdC) in Charleroi. Patient selection was made by using the hospital pharmacy list of all patients who received T-DM1. In total, 51 patients were included in the analysis: 18 at CUSL, 19 at CMSE and 14 at GHdC.

The study was approved by the ethics committees of all participating institutions. As this was a retrospective study, informed consent was not required.

### Outcome assessment

The aim of the study was to evaluate PFS during treatment with T-DM1 based on the line of treatment in which it was given. For the purposes of this analysis, we defined PFS as the period between the first administration of the treatment and the first clinical or radiological examination that showed a progression of the cancer. The evaluation of the disease was performed every 12 weeks (usually by CT-scan, with the addition of brain magnetic resonance imaging if clinically indicated) in each center.

T-DM1 was generally used in second line, after the association of a taxane and trastuzumab. If there was an early relapse of the disease (during neoadjuvant treatment or the 6 following months), T-DM1 was usually used in first line. In some cases, T-DM1 was administered in later lines due to drug unavailability at the time of relapse.

As T-DM1 is usually administered in the first lines and not in later lines, we planned to compare the PFS of T-DM1 in the first 3 lines vs later and in the first 2 lines vs later, which allows for larger groups of patients.

We also evaluated if pre-treatment with pertuzumab or capecitabine-lapatinib has an impact on PFS under T-DM1.

### Statistical analysis

Statistical analysis and graphics were performed using SAS9.4 software. A *p*-value less than 0.05 was considered as statistically significant. PFS rates were determined by the Kaplan Meier method (Log-Rank Test). All analyses were performed in intention to treat (ITT).

## Results

### Patients’ characteristics

We defined 4 subgroups according to the line of treatment in which T-DM1 was administered. Out of the 51 patients included in the analysis, 4 patients belonged to group 1 (T-DM1 in the first line), 20 to group 2 (T-DM1 in the second line), 11 to group 3 (T-DM1 in the third line), and finally 16 to group 4 (T-DM1 in fourth line or beyond).

Of note, while the vast majority of patients had metastatic disease, 2 patients were treated with T-DM1 for locally advanced inoperable disease.

The clinical and histological features of the study population are summarized in Table 1.

**Table 1:** Clinical and histological features of the study population

Characteristics	First line (*n* = 4)	Second line (*n* = 20)	Third line (*n* = 1)	Fourth line and beyond (*n* = 16)	Total (*n* = 51)
Sex					
F	4 (100%)	20 (100%)	11 (100%)	16 (100%)	51 (100%)
M	0 (0%)	0 (0%)	0 (0%)	0 (0%)	0 (0%)
Age of onset of cancer (median)	56.9 years	52.4 years	50 years	52.9 years	52.9 years
Metastatic from the outset					
Yes	0 (0%)	4 (20%)	3 (27%)	9 (56%)	16 (31%)
No	4 (100%)	16 (80%)	8 (73%)	7 (44%)	35 (69%)
Age of onset of advanced disease	69 years	61 years	50 years	53 years	56 years
Death (median age)					
Yes	2 (50%)(72.9 years)	9 (45%)(64.9 years)	3 (27%)(47.4 years)	7 (44%)(63.7 years)	21 (41%)(64.9 years)
No	2 (50%)	11 (55%)	8 (73%)	9 (56%)	30 (59%)
Histology					
Ductal	3 (75%)	18 (90%)	11 (100%)	16 (100%)	48 (94%)
Lobular	0 (0%)	0 (0%)	0 (0%)	0 (0%)	0 (0%)
Mixed (ductal and lobular)	1 (25%)	1 (5%)	0 (0%)	0 (0%)	2 (4%)
ND	0 (0%)	1 (5%)	0 (0%)	0 (0%)	1 (2%)
Grade					
I	1 (25%)	0 (0%)	0 (0%)	0 (0%)	1 (2%)
II	2 (50%)	6 (30%)	8 (73%)	5 (31%)	21 (41%)
III	1 (25%)	9 (45%)	1 (9%)	10 (63%)	21 (41%)
II-III	0 (0%)	2 (10%)	0 (0%)	1 (6%)	3 (6%)
ND	0 (0%)	3 (15%)	2 (18%)	0 (0%)	5 (10%)
ER, PR^a^					
+/+	2 (50%)	5 (25%)	5 (45%)	10 (62%)	22 (43%)
−/−	2 (50%)	9 (45%)	2 (18%)	2 (12%)	15 (29%)
Other = +/−, −/+	0 (0%)	5 (25%)	4 (36%)	4 (25%)	13 (25%)
ND	0 (0%)	1 (5%)	0 (0%)	0 (0%)	1 (2%)
Visceral metastases at diagnostic of advanced disease					
+	1 (25%)	15 (75%)	9 (82%)	11 (69%)	36 (71%)
-	3 (75%)	5 (25%)	2 (18%)	5 (31%)	15 (29%)
SNC metastasis at diagnostic of advanced disease					
+	0 (0%)	5 (25%)	2 (18%)	0 (0%)	7 (14%)
-	4 (100%)	15 (75%)	9 (82%)	16 (100%)	44 (86%)
Pretreatment by taxane-trastuzumab-pertuzumab					
+	0 (0%)	14 (70%)	2 (18%)	1 (6%)	17 (33%)
-	4 (100%)	6 (30%)	9 (82%)	15 (93%)	34 (67%)
Pretreatment by capecitabine-lapatinib					
+	0 (0%)	2 (10%)	5 (45%)	5 (31%)	12 (24%)
-	4 (100%)	18 (90%)	6 (55%)	11 (69%)	39 (76%)

^a^negativity if cell tagging < 1% by immunohistochemistry

Abbreviations: *ER* Estrogen receptor, *F* Female, *M* Male, *NA* Not applicable, *ND* Not determined, *PR* Progesterone receptor

There were differences between the treatment groups in terms of proportion of positive hormonal status of the tumor, grade of the tumor, proportion of patients with metastases from outset of the disease, age at onset of advanced disease and proportion of visceral metastases at diagnosis of advanced disease, but few metastasis in the SNC. Seventeen patients were pretreated with the combination taxane-trastuzumab-pertuzumab in the metastatic setting, which was every time given just before the administration of T-DM1 (median time between progressive disease under pertuzumab and start of T-DM1 was 22 days), mainly in first line (15 patients).

### Progression-free survival across all lines

**Fig. 1:**
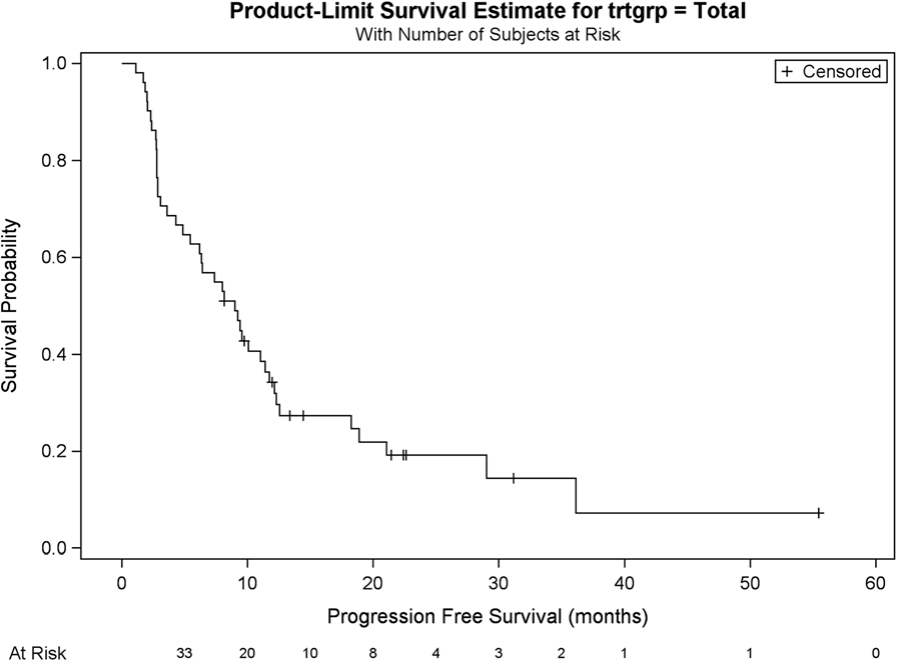
PFS across all lines of treatment with T-DM1

At the time of the analysis, 10 patients had not progressed under T-DM1 (19.6%), while 41 patients had shown progressive disease (80.4%). The median PFS is 9.01 months, ranging from 1.1 to 55.5 months. After 1 year of follow-up, 34.3% of patients had not progressed under T-DM1. At 4 years, this percentage had decreased to 7.2%, as shown in Table 2 and Fig. 1.

**Table 2:** PFS across all lines of treatment with T-DM1

Characteristics		Total (*n* = 51)
Progression	No	10 (19.6%)
	Yes	41 (80.4%)
PFS according to Kaplan-Meier (months)	Median	9.01
	CI95	(4.87–11.41)
	Minimum	1.1
	Maximum	55.5
PFS rate	1 year	34.3%
	2 years	19.2%
	3 years	14.4%
	4 years	7.2%

### Progression-free survival according to the line of treatment

We analyzed the influence of the line of treatment on PFS and found that the line of treatment in which T-DM1 is administered does not appear to influence PFS (Hazard Ratio 0.976 and CI95 0.835–1.142).

### Progression-free survival, lines 1–3 vs 4–10

**Fig. 2:**
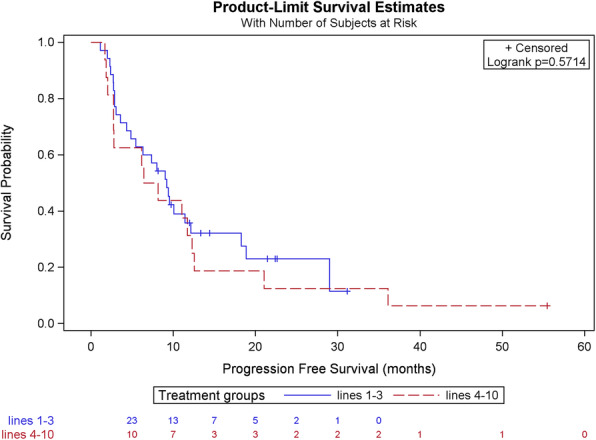
Comparison of PFS in patients receiving T-DM1 in lines of treatment 1–3 as compared to 4–10

We compared the PFS of patients treated with T-DM1 in lines 1 to 3 with the PFS of more heavily pretreated patients, treated with T-DM1 in lines 4 to 10. We found no statistically significant difference between both groups, as the median PFS was 9.21 months for lines 1 to 3 and 7.29 months for lines 4 to 10 (Logrank test *p*-value 0.571) (see Table 3 and Fig. 2).

**Table 3:** Comparison of PFS in patients receiving T-DM1 in lines of treatment 1–3 as compared to 4–10

Characteristics	Lines 1–3 *n* = 35	Lines 4–10 *n* = 16	Total *n* = 51
Progression			
No	9 (25.7%)	1 (6.3%)	10 (19.6%)
Yes	26 (74.3%)	15 (93.8%)	41 (80.4%)
PFS according to Kaplan-Meier (months)			
Median	9.21	7.29	9.01
CI95	(4.87–12.14)	(2.73–12.30)	(4.87–11.41)
Minimum	1.1	1.7	1.1
Maximum	31.2	55.5	55.5
Intergroup comparison (Log-Rank test)			
*p*-value			0.571
PFS rate			
1 year	35.7%	31.3%	34.3%
2 years	23.0%	12.5%	19.2%
3 years	NA	12.5%	14.4%
4 years	NA	6.3%	7.2%

### Progression-free survival, lines 1–2 vs 3–10

**Fig. 3:**
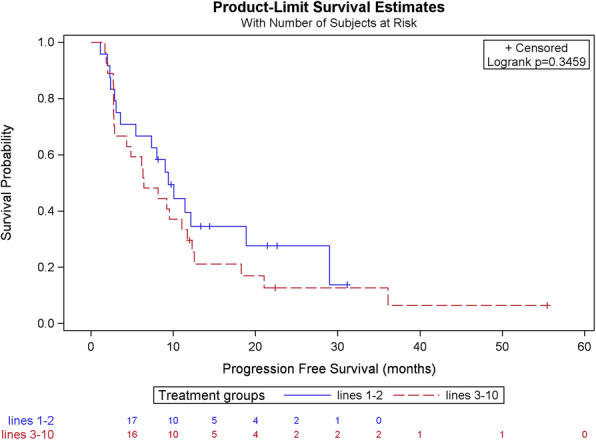
Comparison of PFS in patients receiving T-DM1 in lines of treatment 1–2 as compared to 3–10

We then decided to use a different cut-off to define even less pretreated patients, having received only 1 or 2 lines of therapy, which we compared with the patients who had received from 3 to 10 lines of therapy. When treated with T-DM1, the median PFS of lines 1–2 is 9.41 months, as compared to 6.41 months for lines 3–10. The difference between these two groups is also not statistically significant (*p*-value 0.346), as shown in Table 4 and Fig. 3.

**Table 4:** Comparison of PFS in patients receiving T-DM1 in lines of treatment 1–2 as compared to 3–10

Characteristics	Lines 1–2 *n* = 24	Lines 3–10 *n* = 27	Total *n* = 51
Progression			
No	7 (29.2%)	3 (11.1%)	10 (19.6%)
Yes	17 (70.8%)	24 (88.9%)	41 (80.4%)
PFS according to Kaplan-Meier (months)			
Median	9.41	6.41	9.01
CI95	(3.59–18.88)	(2.80–11.74)	(4.87–11.41)
Minimum	1.1	1.7	1.1
Maximum	31.2	55.5	55.5
Intergroup comparison (Logrank test)			
*p*-value			0.346
PFS rate			
1 year	35.7%	29.6%	34.2%
2 years	35.7%	12.7%	19.2%
3 years	11.5%	12.7%	14.4%
4 years	NA	6.4%	7.2%

### Progression-free survival as a function of prior treatment with capecitabine and lapatinib

**Fig. 4:**
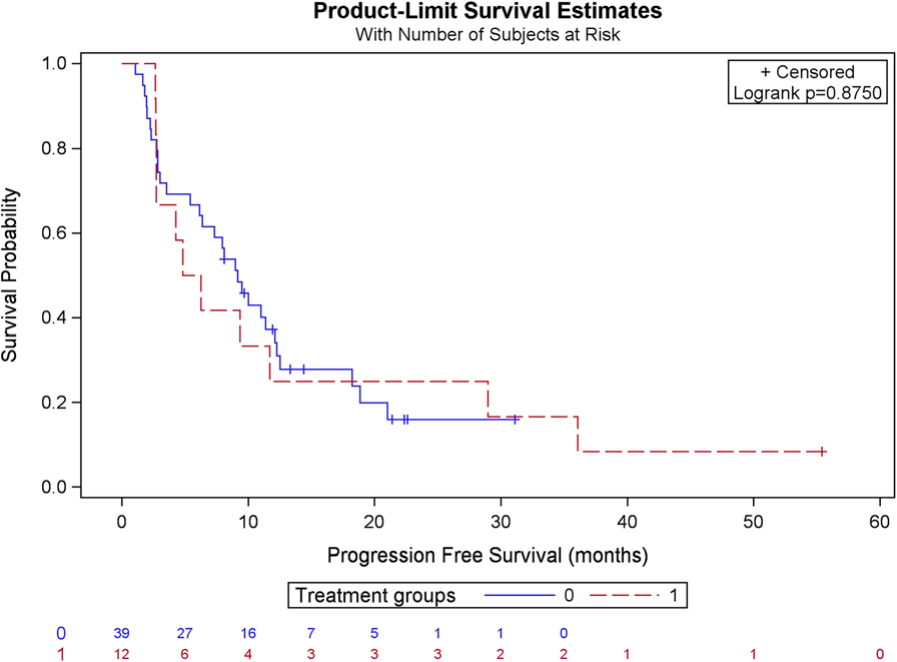
PFS in patients receiving T-DM1 with or without prior treatment with capecitabine and lapatinib

We analyzed the PFS of our patients according to prior exposure to capecitabine and lapatinib (Table 5 and Fig. 4). Patients who had not been pretreated with the combination capecitabine and lapatinib had a median PFS of 9.17 months, as compared to 5.56 months in pretreated patients. This difference was not statistically significant (*p*-value 0.875).

**Table 5:** Comparison of PFS in patients receiving T-DM1 with or without prior treatment with capecitabine and lapatinib

Characteristics	No prior capecitabine and lapatinib *n* = 39	Prior capecitabine and lapatinib *n* = 12	Total *n* = 51
Progression			
No	9 (23.1%)	1 (8.3%)	10 (19.6%)
Yes	30 (76.9%)	11 (91.7%)	41 (80.4%)
PFS according to Kaplan- Meier (months)			
Median	9.17	5.56	9.01
CI95	(5.42–12.10)	(2.70–28.96)	(4.87–11.41)
Minimum	1.1	2.7	1.1
Maximum	31.1	55.4	55.5
Intergroup comparison (Log-Rank test)			
*p*-value			0.875

### Progression-free survival as a function of prior treatment with taxane-trastuzumab-pertuzumab

**Fig. 5:**
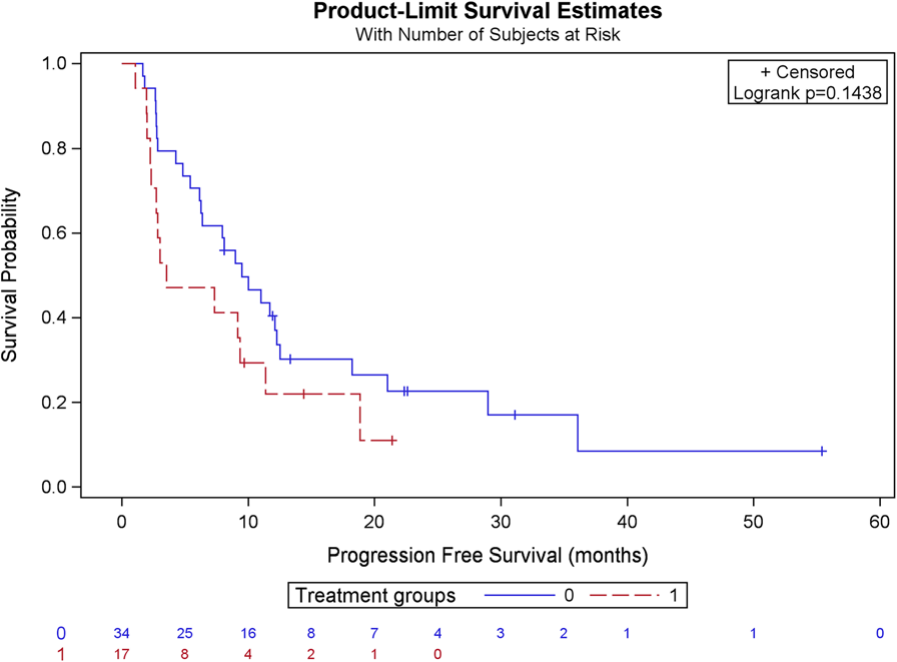
PFS in patients receiving T-DM1 with or without prior treatment with pertuzumab

We then went on to compare the PFS of patients who had not been treated with the association taxane-trastuzumab-pertuzumab prior to receiving TDM-1 with the PFS of patients who had (Table 6 and Fig. 5). The median PFS was 9.50 months in patients with no prior exposure to taxane-trastuzumab-pertuzumab and 3.55 months in patients treated with T-DM1 after this combination. There was thus a trend towards a lower PFS in patients who had prior exposure to taxane-trastuzumab-pertuzumab, which was however not statistically significant (*p*-value 0.144).

**Table 6:** Comparison of PFS in patients receiving T-DM1 with or without prior treatment with taxane-trastuzumab-pertuzumab

Characteristics	No prior pertuzumab *n* = 34	Prior pertuzumab *n* = 17	Total *n* = 51
Progression			
No	7 (20.6%)	3 (17.6%)	10 (19.60%)
Yes	27 (79.4%)	14 (82.4%)	41 (80.4%)
PFS according to Kaplan-Meier (months)			
Median	9.50	3.55	9.01
CI95	(6.15–12.26)	(2.27–11.38)	(4.87–11.41)
Minimum	1.7	1.1	1.1
Maximum	55.4	21.4	55.5
Intergroup comparison (Log-Rank test)			
*p*-value			0.144

## Discussion

Based on our results, the line of treatment in which T-DM1 is administered for metastatic or unresectable locally advanced breast cancer has no impact on PFS, unlike what is generally observed with standard chemotherapy, where PFS usually declines with advancing lines of treatment [6]. In our unselected patient population, the median PFS of 9.01 months (CI95 4.87–11.41) was consistent with the 9.6 months observed in the Emilia trial [3]. Nevertheless, some results in the literature contradict this finding. The KAMILLA study, a phase IIIb, one-arm, international, open-label study evaluated safety and tolerability (primary endpoints) and OS/PFS (secondary endpoints) of T-DM1 in advanced HER2-positive breast cancer patients pretreated with any chemotherapy and an anti-HER2 agent [10]. This post-marketing study included 2002 patients of which 1321 had already received two prior lines of therapy. It found a median PFS of 6.9 months but also that PFS decreases with increasing prior lines of treatment (from 8.3 months in the first and second lines of treatment to 5.6 months in 4+ lines of treatment). The KAMILLA study is the largest cohort of patients treated with T-DM1 and is more representative of real-life data as it included more patients with symptomatic disease, positive hormonal status and visceral metastases than the Emilia trial.

As patients included in the Emilia trial could not have been pretreated with capecitabine and lapatinib (since this was the comparator arm to T-DM1), data on the efficacy of T-DM1 in this patient population are scarce. Because a significant proportion of our cohort had already progressed on capecitabine and lapatinib once T-DM1 became commercially available, we had the opportunity to assess whether prior treatment with these compounds had an impact on PFS with T-DM1. Reassuringly, our data do not suggest any such impact. When the combination of capecitabine and lapatinib was administered before T-DM1, the PFS was 5.56 months (CI95: 2.70–28.96), as compared to 9.17 months (CI95: 5.42–12.10) when this was not the case (*p* = 0.875). This is consistent with the results of the Th3RESA trial which compared T-DM1 to physician’s choice of treatment, for advanced breast cancers pretreated with at least 2 lines of treatment including trastuzumab, lapatinib and a taxane. In this study, median PFS was 6.2 months for T-DM1 vs 3.3 months for physician’s choice treatment (HR 0.528 (CI95: 0.422–0.661)).

On the contrary, while not statistically significant, in our study, PFS tended to be shorter when pertuzumab had been given before T-DM1 (9.50 months if no prior pertuzumab, vs 3.55 months in case of prior exposure to pertuzumab, *p* = 0.144). In the EMILIA study, which compared the administration of lapatinib plus capecitabine to the administration of T-DM1, the number of patients who received pertuzumab prior to receiving T-DM1 was very limited. However, since the publication of the Cleopatra study, the standard first-line treatment is a combination of taxanes, trastuzumab and pertuzumab.

A study conducted in 2016 demonstrated the efficacy of T-DM1 for patients who had received prior pertuzumab [5]. The authors of this study, in which all patients had been treated with pertuzumab before receiving T-DM1, showed that 31% of these patients exhibited a PFS greater than 6 months. The mean duration of therapy with T-DM1 was 4 months, which is similar to our median PFS of 3.55 months with T-DM1 in pertuzumab pretreated patients.

Several other observational studies or registries evaluated PFS with T-DM1 after taxane-trastuzumab-pertuzumab with different results. In the GIM14/BIOMETA study [11], a retrospective/prospective multicenter study on treatment regimens in metastatic breast cancer, 77 patients received T-DM1 in second line after taxane-trastuzumab-pertuzumab, with a median PFS of 6.3 months, which is longer than what we observed in our cohort. In the PRAEGNANT metastatic breast cancer registry, 76 patients were treated with T-DM1 in different lines after taxane-trastuzumab-pertuzumab (39 in second-line, 25 in third-line and 12 in fourth-line or higher). This study found a median PFS with T-DM1 of 3.5 months in all patients but with a decline of PFS when T-DM1 was administered in later lines (median PFS of 7.7 months in second-line, 3.4 months in third-line and 2.7 months in fourth-line or higher) [12].

Finally, in a retrospective observational study conducted in 250 patients in 23 centers, the median PFS with T-DM1 was not modified by pretreatment with pertuzumab (median PFS 4 months in pretreated patients and 6 months in pertuzumab-naïve patients) [13].

It is interesting to note that the median PFS in our study among patients pretreated with pertuzumab is numerically lower than in patients not pretreated with pertuzumab, although this is not statistically significant, probably because of the insufficient number of patients included in our study.

Finally, we could point out some important limitations to our study. It is a retrospective study, with a limited number of patients included, particularly in the group of patients treated with T-DM1 in first line for advanced disease (4 patients).

## Conclusions

Our study suggests that T-DM1 remains active in all lines of treatment of HER2 positive metastatic and locally advanced breast cancer. PFS does not seem to be impacted by prior treatment with capecitabine and lapatinib, but there is a trend toward reduced PFS in patients who progressed on prior treatment with pertuzumab.

Nevertheless, this is a small retrospective study with limited data, unlike the KAMILLA study for example, which shows a decline in PFS with increasing lines of treatment.

For this reason, our findings should be confirmed with stronger data, such as a large registry of real-world data.

## Data Availability

The datasets used and/or analyzed during the current study are available from the corresponding author on reasonable request.

## Abbreviations

CMSE: CHU UCL Namur (site Sainte-Elisabeth), Namur, Belgium
CUSL: Cliniques Universitaires Saint-Luc, Brussels, Belgium
ER: Estrogen receptor
F: Female
GHdC: Grand Hôpital de Charleroi, Charleroi, Belgium
HER2: Human epidermal growth factor receptor 2
M: Male
NA: Not applicable
ND: Not determined
PFS: Progression-free survival
PR: Progesterone receptor
T-DM1: Trastuzumab emtansine
